# Targeting highly attenuated IL-18 to PD-1 for enhanced anti-tumor activity

**DOI:** 10.3389/fimmu.2025.1718321

**Published:** 2025-12-18

**Authors:** Xueyuan Zhou, Felix Klaus Geyer, Jeffrey Takimoto, Harald Kolmar, Brian Rabinovich

**Affiliations:** 1 Drug Discovery and Development, Fuse Biotherapeutics, Woburn, MA, United States; 2 Institute for Organic Chemistry and Biochemistry, Technical University of Darmstadt, Darmstadt, Germany; 3 Centre for Synthetic Biology, Technical University of Darmstadt, Darmstadt, Germany

**Keywords:** checkpoint inhibitor, cytokine, IL-18, immunocytokine, PD-1

## Abstract

Checkpoint inhibitors targeting the PD-1/PD-L1 axis have revolutionized cancer immunotherapy, yet response rates remain limited. To enhance efficacy, next-generation approaches target T cell-activating cytokines to PD-1 via antibodies. The goal is simultaneous checkpoint blockade and cytokine potentiation but fine-tuning cytokine activity such that checkpoint inhibition can be preserved with manageable toxicity has been a difficult challenge. We hypothesized that targeting a highly attenuated interleukin (IL)-18 to PD-1 can activate PD-1+ T cells and oppose exhaustion while antagonizing PD-1. We generated a highly attenuated IL-18 variant, which is resistant to IL-18BP binding and assessed its receptor binding ability. Tumor growth inhibition was evaluated across multiple models. Additionally, we examined post-remission tumor resistance and lymphocyte infiltration into the tumor ex vivo using flow cytometry. The IL-18 fusion resisted interleukin-18 binding protein (IL-18BP) inhibition and exhibited a 10,000-fold reduction in activity while preserving cis-signaling and demonstrated strong efficacy across tumor models. It increased CD8+ progenitor-exhausted tumor-infiltrating lymphocytes (TILs) while reducing myeloid TILs. Attaching a highly attenuated IL-18 to an anti-PD-1 antibody goes beyond simply targeting a cytokine to PD-1, representing a novel cytokine-enhanced checkpoint inhibitor that activates PD-1+ T cells via the cytokine receptor while simultaneously antagonizing PD-1.

## 1 Introduction

PD-1 is an inhibitory receptor expressed on activated/exhausted T cells, B cells, NK cells and myeloid cells (1). Its ligand, PD-L1, is found on various cell types, including lymphocytes, epithelial cells, and tumor cells (2). The PD-1/PD-L1 interaction plays a crucial role in maintaining immune balance and preventing autoimmunity by acting as a checkpoint to prevent uncontrolled T cell activity (3). This regulatory axis is often active in tumors, where PD-L1 promotes an immunosuppressive environment (2). Activated or exhausted lymphocytes upregulate PD-1 in response to neoantigens — mutated gene products absent during T cell education (4). To counteract tumor PD-L1 expression, PD-1/PD-L1 inhibitors were developed to reactivate suppressed lymphocytes (5). Although αPD-1 can antagonize PD-1 on exhausted T cell populations ranging from the progenitor exhausted (TpEX) to terminally exhausted (TtEX), rejuvenation of TpEX has been reported as responsible for the anti-tumor activity ascribed to PD-1/PD-L1 blockade (6). As such, once terminal exhaustion is reached, αPD-1 is no longer effective, resulting in resistance to therapy (7). Despite the success of PD-1/PD-L1 antagonists, the vast majority of cancer patients are either innately resistant (up to 60% of patients) or acquire a refractory phenotype during treatment, resulting in durable responses in only about 20% of treatment eligible patients (8–10).

A promising approach to enhancing the activity of αPD-1 is fusing T cell activating cytokines to PD-1 targeted antibodies. The overarching goal of our work is to provide PD-1 positive T cells with a signal that promotes expansion and survival, while maintaining checkpoint inhibition. Several cytokines including IL-2 (11, 12), IL-15 (13, 14), IL-12 (15, 16), and IL-18 (17) have been fused to either PD-1 or PD-L1 targeting antibodies. IL-18 is of particular interest because of its ability to induce the expression of T-bet, reported to oppose terminal exhaustion (4, 18). Other advantageous properties include induction of anti-apoptotic proteins including Bcl-xL (19), granzymes (20), IFNγ (21), proliferation (22), effector memory (23) and opposition of TGFβ induced fibrosis (24). Importantly, within tumor infiltrating lymphocytes (TILs), the IL-18 receptor complex (IL-18RC) has been reported as preferentially expressed on PD-1+ TpEX (25, 26) meaning that delivery of IL-18 to these cells may induce their preservation/expansion, increase their cytotoxicity and inhibit their terminal exhaustion. To deliver IL-18 to PD-1+ target cells, two hurdles must be overcome. The first is overcoming inhibition by IL-18’s natural antagonist, IL-18BP (27), which is induced by IFNγ and has a higher affinity for IL-18 than the IL-18 receptor complex. Mutations can be introduced into IL-18 that resist binding to IL-18BP (28), but once added the need for precision targeting of IL-18 with little to no systemic activity becomes paramount to avoid toxicity mediated by activation of cells outside the TME including macrophages, NK cells, vascular endothelial cells, vascular smooth muscle cells (29, 30). IL-18 is a danger-induced alarmin that is translated as a pro-cytokine containing an endogenous propeptide that silences IL-18 in the producing cell through a disulfide bridge until it is processed by Caspase-I and released into the extracellular space. IL-18 binds first to IL-18Rα, for which it has a moderate binding affinity of ~20–40 nM and then recruits IL-18Rβ, for which it has negligible affinity alone, leading to the formation of a strong trimeric complex (K_D_ ~ 1nM) that is required for signaling (31–33).

Since IL-18 is a highly potent molecule we went out to identify IL-18 variants with highly attenuated potency that when fused to an PD-1 targeting antibody on T cells provide an appropriate activity/toxicity window. By application of protein engineering methods (data not shown) we identified a variant of IL-18, attenuated ~10,000-fold, which, when fused to an αPD-1 antibody, enhances anti-tumor activity and preserves/expands TpEX cells in the tumor microenvironment while maintaining full checkpoint inhibition with minimal systemic activity. Herein we describe the discovery of a drug candidate that delivers IL-18 signaling in cis with promising functional properties *in vivo*.

## 2 Materials and methods

### 2.1 Vector construction and recombinant protein preparation

Anti-mouse PD-1 clone RMP1–14 was used for all IL-18 variants targeting mouse PD-1. Codon-optimized DNA constructs synthesized by BioIntron were cloned into the pCDNA3.4 IgG vector (CMV promoter) with effector-null mutations (L234A, L235A) in the heavy chain. For antibody-IL-18 fusions, attenuated IL-18 variants were fused to one heavy chain’s N-terminus with Hole mutations (S354C, T366S, L368A, Y407V), while the other carried Knob mutations (Y349C, T366W) for Fc heterodimerization. RF mutations (H435R, Y436F) in the Hole chain reduced Hole/Hole homodimer Protein A binding. Constructs were sequence-verified and transfected into ExpiCHO cells (Thermo Fisher, Cat No. A14527). Cells (6.0×10^6^) were mixed with 3.5 mL homemade BioIntron electrolysis solution and plasmid, electroporated, and cultured in 100 mL OPM medium (Cat No. P93059) at 37°C, 120 rpm, 8% CO_2_. Sodium butyrate was added after 24 hours, and culture continued for 6 days. Recombinant proteins were purified via Protein A chromatography (VDOBIOTECH, Cat No. HQ320827001L) and assessed for monomer purity via SEC. Samples <95% monomer purity underwent additional SEC on a Superdex 200 Increase column (Cytiva, Cat No. 17104302) using an ÄKTA pure system (Cytiva, Cat No. 29018226). Monomeric fractions were pooled and formulated in 20 mM sodium citrate, 50 mM NaCl, 3% mannitol, 20 mM DTPA, 0.01% polysorbate 80, pH 6.0.

### 2.2 Mice

C57BL/6 mice were sourced from Gempharmatech (Nanjing, Jiangsu, China). BALB/c mice were obtained from Biocytogen (Beijing, China). Animal studies were conducted at various contract research organizations (CROs), including Kyinno Biotechnology (Beijing), Innomodels Biotechnology (Beijing), Medicilon (Shanghai), and Viva Biotech (Shanghai). Animals were housed under standard conditions (22°C, 50% humidity, 12-hour light/dark cycle, unlimited sterilized water and food) at each CRO’s facility, adhering to animal welfare guidelines and approved protocols from each CRO’s animal care and use committee. Mice were euthanized using CO_2_ inhalation at a displacement rate of 30–70% chamber volume per minute (e.g., 3–7 L/min in a 10-L chamber). For retro-orbital blood collection, mice were anesthetized with ketamine (90–120 mg/kg) plus xylazine (8–12 mg/kg) administered intraperitoneally, and a topical ophthalmic anesthetic (proparacaine) was applied to minimize discomfort.

### 2.3 Cell lines

HEK-Blue-IL-18 cells (Invivogen, Cat No: hkb-hmil18) were cultured in DMEM medium (ThermoFisher, Cat No: 12430-047) supplemented with 10% heat-inactivated fetal bovine serum (FBS) (ThermoFisher, Cat No: 10082147), 1× non-essential amino acids (ThermoFisher, Cat No: 11140050), 1× Sodium pyruvate (ThermoFisher, Cat No: 11360070), 1× penicillin-streptomycin (Fisher Scientific, Cat No: 15070063), 100 μg/mL Normocin (Invivogen, Cat No: ant-nr-1), and 1× HEK-blue selection reagent (Invivogen, Cat No: hb-sel). MDA-MB-231 eGFP Fluc cells (human ROR1-positive, GeneTarget, Cat No: SC-044) were grown in DMEM/F12 medium (ThermoFisher, Cat No: 11330-032) with the same supplements. MC38i cells, an aggressive MC38 clone, were used by Innomodels Biotechnology (Beijing). MC38 cells were also sourced from Kyinno Biotechnology (Beijing, Cat No: KC-1279) and Medicilon (Shanghai). B16-F10-hROR1 cells, a B16-F10 clone expressing human ROR1, were from Kyinno (Cat No: KC-0526-JD). These cells were maintained in DMEM with 10% FBS, 1× non-essential amino acids, 1× Sodium pyruvate, and 1× penicillin-streptomycin. CT26 cells (Cat No: KC-2162) and CT26.hCDH17 cells (CT26.Q1286/CT26.C, Cat No: KC-3016), both owned by Kyinno, were cultured in RPMI1640 medium (Gibco, Cat No: 22400121) with the same supplements.

### 2.4 Mouse splenocyte isolation

Mouse splenocytes were isolated by placing the spleen in a petri dish (ThermoFisher, Cat No: 150466) with 5 mL RPMI1640 complete medium and rinsing twice with RPMI1640. The spleen was mashed through a 100-µm cell strainer (Corning, Cat No: 352360) placed in a new petri dish with 3 mL RPMI1640, using the plunger of a 5-mL syringe (Fisher Scientific, Cat No: 14955458) to create a single-cell suspension. The suspension was transferred to a 50-mL conical tube (Fisher Scientific, Cat No: 14-955-240). The strainer was rinsed with RPMI1640, and the rinse was combined with the cells. The mixture was centrifuged at 500× g for 5 minutes, resuspended in 5 mL 1× ACK Lysis buffer (ThermoFisher, Cat No: 00-4300-54) for 5 minutes, then diluted with 45 mL RPMI1640 complete medium and centrifuged again. The splenocytes were resuspended for immediate use or frozen in liquid nitrogen.

### 2.5 Mouse splenocyte activation assay

Fresh mouse splenocytes were resuspended in RPMI1640 complete medium at 1 million cells/mL. Rat anti-mouse CD3 antibody clone 145-2C11 (Biolegend, Cat No: 100302) was added at 1 µg/mL, and cells were incubated at 37°C, 5% CO_2_ for 48 hours. After incubation, cells were washed twice with RPMI1640 by centrifuging at 500 × g for 5 minutes and resuspended in medium containing 100 ng/mL anti-CD3 antibody at 1.334 million cells/mL. 150 µL of the suspension (0.2 million cells/well) was added to 96-well plates, and 150 µL of testing articles at 2× concentration was added. Cells were incubated for 24–120 hours at 37°C, 5% CO_2_. Supernatants were collected for IFNγ detection using the mouse IFNγ ELISA kit (Biolegend, Cat No: 430816) per the manufacturer’s protocol.

### 2.6 Targeted mouse splenocyte activation assay

MDA-MB-231 eGFP Fluc cells were collected using TrypLE (ThermoFisher, Cat No: 12605028), washed with 1× PBS, and resuspended in DMEM/F12 medium with 10% FBS, 1× non-essential amino acids, 1× sodium pyruvate, and 1× penicillin-streptomycin. 100 µL of cell suspension (5000 cells) was added to each well of a 96-well plate (ThermoFisher, Cat No: 165305) and incubated for 4 hours at 37°C, 5% CO_2_. Mouse splenocytes were resuspended in RPMI1640 medium at 2 million cells/mL. Human ROR1×mouse CD3 bispecific antibody was prepared in RPMI1640 at 4× concentration, and testing articles in RPMI1640 at 2× concentration. After 4 hours, the medium was removed from target cells, and 75 µL of splenocyte suspension (150,000 cells) was added to each well, along with 75 µL bispecific antibody and 150 µL testing article. Plates were incubated for 72 hours at 37°C, 5% CO_2_, and supernatants were collected for IFNγ detection using the mouse IFNγ ELISA kit (Biolegend, Cat No: 430816).

### 2.7 IFNγ release detection by ELISA

Mouse IFNγ was detected using the mouse IFNγ ELISA kit. 100 µL of diluted coating antibody was added to a Nunc MaxiSorp 96-well plate (ThermoFisher, Cat No: 442404) and incubated at 4°C for 16 hours. Plates were washed four times with wash buffer (1× PBS, 0.02% Tween-20), blocked with 200 µL of 1× assay diluent at room temperature for 2 hours, and washed again. Standards or samples (100 µL) were incubated for 2 hours, followed by washing and adding 100 µL of biotinylated detection antibody for 1 hour. After washing, 100 µL of diluted avidin-HRP solution was added and incubated for 30 minutes. Plates were washed five times, then incubated with 100 µL TMB substrate for 20 minutes in the dark. The reaction was stopped with 100 µL stop solution, and absorbance at 450 nm was measured. IFNγ concentrations were calculated from the standard curve.

### 2.8 Biolayer interferometry

Biolayer interferometric measurements were performed using an Octet RED96 system (ForteBio, Sartorius, Germany). Anti-murine IgG Fc Capture (AMC) biosensors (Sartorius, Cat No: 18-5088), High Precision Streptavidin (SAX) biosensors (Sartorius, Cat No: 18-5117), or anti-His Capture biosensors (Sartorius, Cat No: 18-5120) were soaked in 1× PBS (pH 7.4) for 10 minutes before assay initiation. The instrument was pre-warmed for 30 minutes, and measurements were conducted at 25°C with a shake speed of 1000 rpm. Immobilizing proteins were prepared at 10 µg/mL in PBS, and detection proteins were prepared and serially diluted in kinetics buffer (KB; Sartorius, Germany). Biosensors were first equilibrated in PBS for baseline acquisition, followed by loading of immobilizing proteins until a stable signal was reached. A short quenching step in kinetics buffer (KB) was performed before sensors were exposed to detection proteins for the association phase. Dissociation was monitored in kinetics buffer for the required duration. Data analysis was performed using ForteBio Data Analysis Software 9.0.0.14, and binding kinetics were determined using Savitzky–Golay filtering and a 1:1 Langmuir binding model.

### 2.9 *In-vivo* tumor growth inhibition study

1×10^6^ MC38i, 1×10^6 B16-F10-hROR1 tumor cells were injected subcutaneously (s.c.) into C57BL/6 mice (6–8 weeks). For BALB/c mice, 1×10^6^ CT26 or 3×10^6 CT26.hCDH17 (CT26.C) cells were injected s.c. Mice were grouped when tumors reached 75 mm^3 (small tumors). Testing articles were administered intraperitoneally (i.p.) at 5, 10, or 15 mg/kg on days 0 and 4 or days 0, 4, and 8, depending on the experiment. Tumor size and mouse weight were measured every three days, and tumor volume was calculated as length × width²/2. Mice were sacrificed when tumors reached 4000 mm³ or at experiment termination, per guidelines.

For IFNγ measurement in MC38i experiments, 1×10^6^ MC38i cells were injected s.c. into C57BL/6 mice (6–8 weeks), grouped at 100 mm³ tumor size, and testing articles were administered i.p. at indicated doses. Blood was collected at 0, 24, 72, and 144 hours and stored at -80°C for IFNγ ELISA. For memory MC38i rechallenge, cured mice were kept for 90 days and reinjected with 3×10^5^ MC38i cells in the opposite flank. Controls received 3×10^5^ MC38i cells without prior treatment. Tumors were measured every three days. For memory and epitope spreading, cured CT26.C-inoculated mice were kept for 65 days before receiving 3×10^5 CT26.C cells in the left flank and 1×10^6^ CT26 cells in the right flank. Controls received CT26.C and CT26 cells without prior treatment. Tumors were measured every three days. For tumor-infiltrating lymphocyte phenotyping, testing articles were administered on days 0, 4, and 6. Mice were sacrificed on days 5 and 6, and whole blood, spleens, and tumors were collected. Splenocytes were isolated, and tumors were dissociated using the mouse tumor dissociation kit (Miltenyi Biotec, Cat No: 130-096-730) and gentleMACS™ Dissociator (Miltenyi Biotec, Cat No: 130-093-235). Cells were analyzed by flow cytometry or frozen for future use.

### 2.10 Statistical analysis and reproducibility

All *in vitro* and ex vivo experiments were repeated at least three times on different days, with samples duplicated or triplicated per experiment. Data, analyzed using GraphPad Prism 9 (v9.5.1), are presented as mean ± SEM. Animal study sample sizes (n) represent biological replicates as shown in figures. Statistical significance (p < 0.05) was assessed using unpaired two-tailed Student’s t-test for two-group comparisons and one-way or two-way ANOVA for multiple groups. Statistical parameters are detailed in figures or legends.

## 3 Results

### 3.1 Engineering of IL-18

The initial objective was to engineer IL-18 variants with suitable biophysical properties for genetic fusion to an αPD-1 antibody. A frequent issue in recombinant cytokine therapeutics is aggregation, which complicates purification, reduces developability, increases immunogenicity risk, and decreases activity (34, 35). IL-18 has four surface-exposed cysteines (C38, C68, C76, and C127) in its mature form that potentially can cause intermolecular crosslinking (36). To exclude this potential problem, all four cysteines in mature IL-18 were uniformly substituted for either valine or serine. While valine substitutions retained activity, serine substitutions reduced activity by ~100-fold (**Supplementary Figure 1**). For the mutants described below, the substitutions to valine were used.

**Figure 1:**
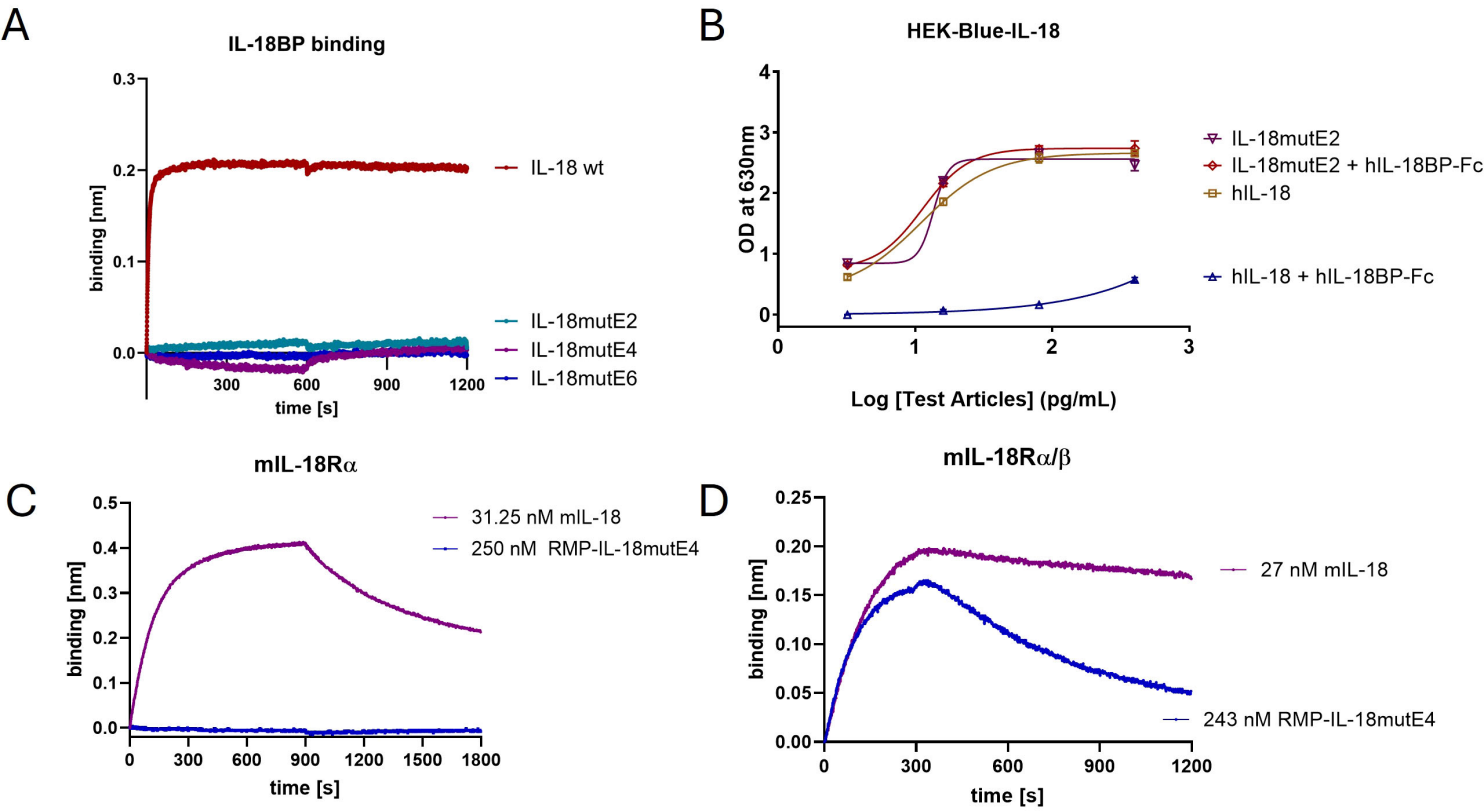
Engineering of IL-18 by incorporation of mutations in the receptor interaction site. **(A)** Binding of 100 nM IL-18mutE2, IL-18mutE4, IL-18mutE6, and IL-18 wild type (wt) to IL-18BP was analyzed by BLI. **(B)** The inhibition of IL-18mutE2 by IL-18BP was also analyzed in a functional HEK-Blue IL-18 reporter cell assay. Upon addition of 1.25 µg/mL IL-18BP-Fc the activity of the wt IL-18 was abolished. **(C)** Investigation of binding of IL-18mutE4 or mIL-18 to mIL-18Rα subunit. **(D)** Investigation of binding of IL-18mutE4 or mIL-18 to the mIL-18Rα/β complex. The corresponding receptor, fused to an Fc domain, was immobilized, and a monovalent IL-18 variant fused to a one-armed anti-mouse PD-1 antibody (RMP-IL-18mutE4) was used as the analyte.

Next, we identified mutants with various degrees of attenuation of murine T cell response called IL-18mut by rational protein design (data not shown) that were considered for *in vivo* studies in mice. To counteract inhibition by IL-18BP, we introduced mutations in IL-18 to reduce its binding to IL-18BP. To this end, mutations were introduced at the interaction site of IL-18 using rational design (**Supplementary Figure 2**). All IL-18 variants displayed binding to IL-18BP that was below the limit of detection of biolayer interferometry (BLI) (**Figure 1A**). Using the HEK-Blue IL-18 reporter cell assay, we confirmed that the IL-18 variants were not inhibited by IL-18BP, while wild type IL-18 showed strong inhibition by IL-18BP (**Figure 1B**). The resulting variants with attenuated potency were named according to the degree of attenuation compared to wild type IL-18 with the suffix E2 for 100-fold, E4 for 10,000-fold, and E6 for 1,000,000-fold attenuation. IL-18mutE4 was engineered based on IL-18mutE2, and IL-18mutE6 was subsequently generated as a further-modified variant of IL-18mutE4. For example, IL-18mutE4 is an IL-18 variant that displays 10,000-fold reduced activity in mice (**Supplementary Figure 3**). No binding of IL-18mutE4 to the murine IL-18Rα subunit was detected by BLI, whereas wild type mIL-18 bound, further confirming that the functional attenuation is mainly due to loss of affinity to IL-18Rα (**Figures 1C, D**).

### 3.2 Fusion of attenuated IL-18 to an αPD-1 antibody enhances anti-tumor activity

**Figure 2:**
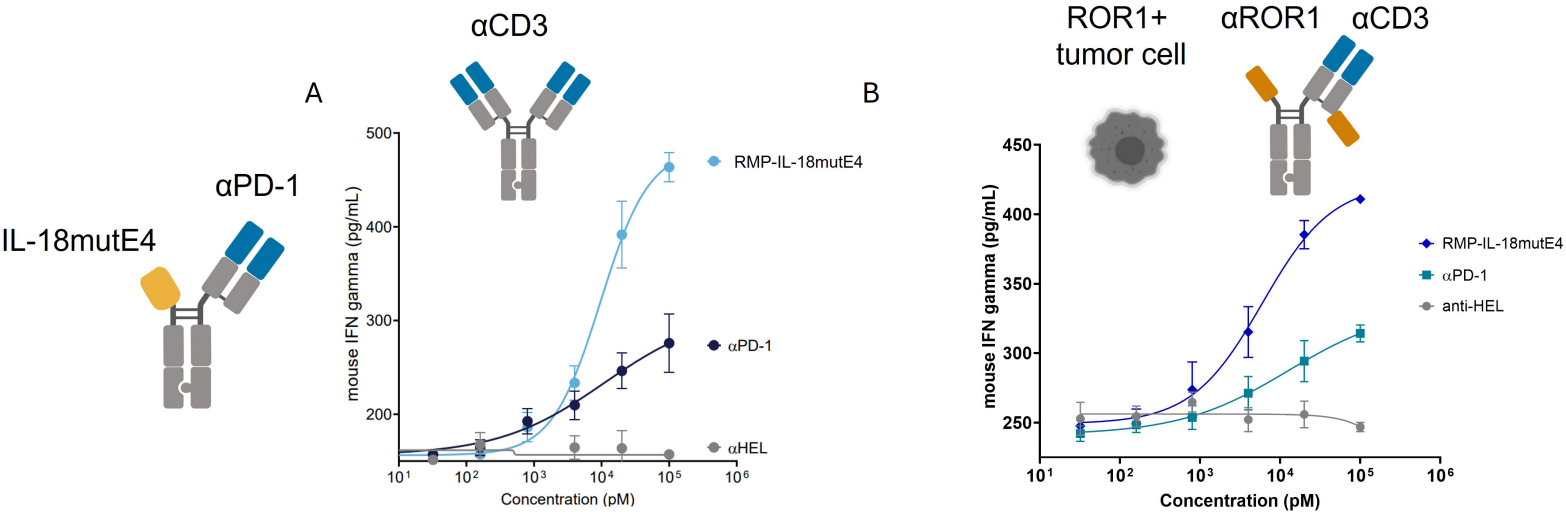
Attenuated IL-18 fused to anti-PD-1 enhances anti-tumor activity. **(A)** Reactivation of αCD3 activated mouse splenocytes resulted in increased activation with RMP-IL-18mutE4 in comparison to αmPD-1 (RMP). Mouse splenocytes were activated with 1 μg/ml αCD3 for 48 h, washed and re-activated with 100 ng/ml αCD3 in the presence of indicated test articles. After 48 h, mIFNγ in the supernatant was measured. **(B)** Mouse splenocytes were cultured with ROR1+ tumor cells plus the 20% maximum activity of a ROR1 x mCD3 TCE at an E:T ratio of 30:1 in the presence of the indicated test articles. IFNγ release was measured after 72 hours. Measurements were performed in duplicates.

IL-18mutE4 was fused to the N-terminus of the heavy chain of a one-armed anti-mouse-PD-1 antibody (RMP1-14 (37)) and assessed in a primary cell system (**Figure 2**). Here, the immunocytokine could not be used alone because mouse T cells require signal 1 to induce expression of the IL-18R complex. Mouse T cells were therefore mixed with either immobilized anti-CD3 (**Figure 2A**) or a combination of ROR1+ MDA-MB-231 and a suboptimal concentration (EC20) of a mouse-CD3 x ROR1 T cell engager (**Figure 2B**) together with the immunocytokine. RMP-IL-18mutE4 enhanced mIFNγ-secretion (~10x EC50, ~2x Emax) from mouse splenocytes activated with anti-CD3, as well as mouse splenocytes co-cultured with ROR1+ tumor cells and redirected to recognize the tumor using the ROR1 T-cell engager (38). Hence, targeting IL-18mutE4 to PD-1+ murine T cells strongly enhanced the activity of PD-1 targeting antibody.

### 3.3 An adequate level of IL-18 attenuation induces strong anti-tumor efficacy but no adverse systemic effects *in vivo*

**Figure 3:**
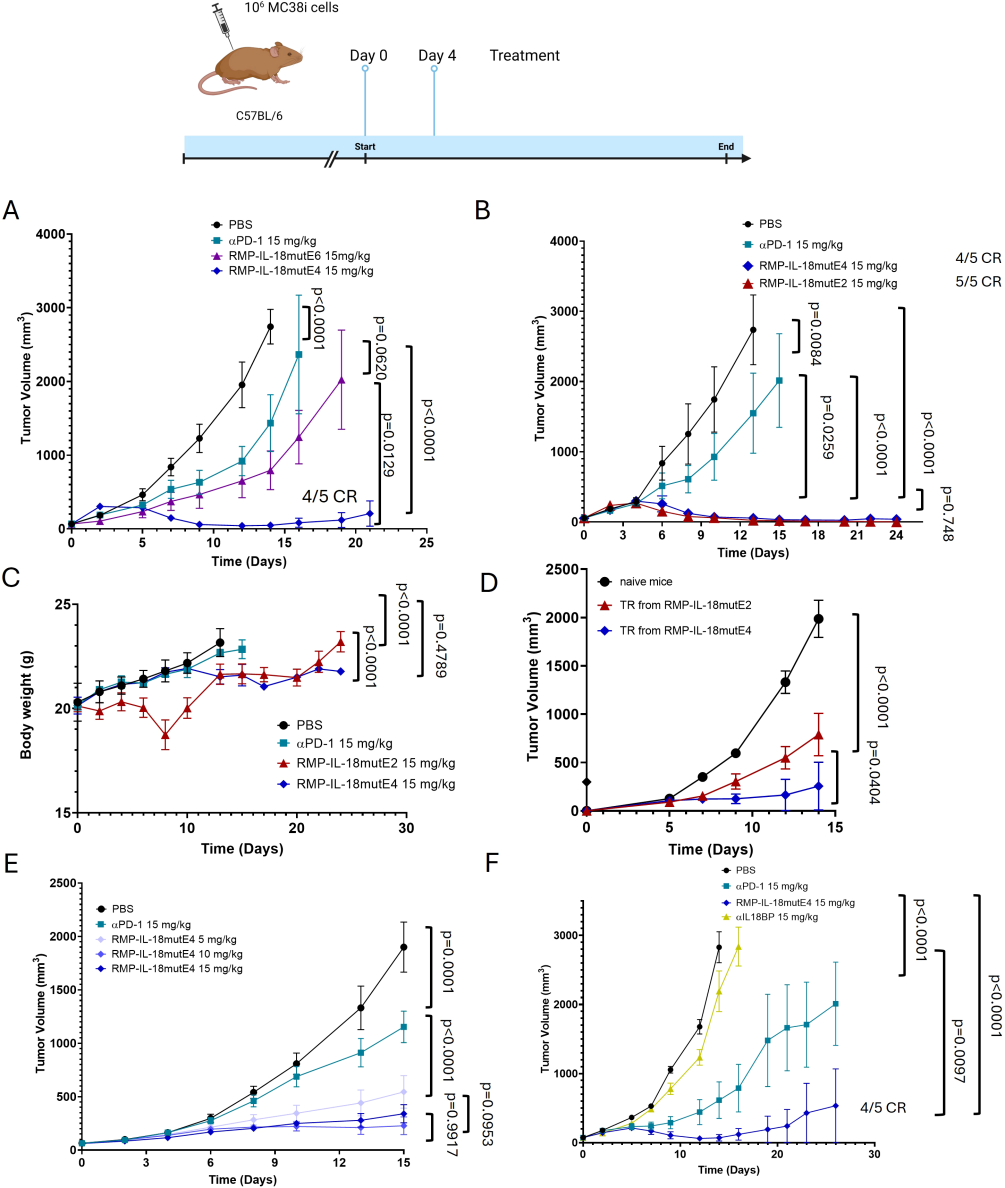
Evaluation of different attenuation degrees of IL-18 in a B6 MC38i tumor model. **(A)** Comparison of 1,000,000-fold (RMP-IL-18mutE6) attenuated IL-18 and the 10,000-fold attenuated IL-18 (RMP-IL-18mutE4) fused to an one-armed αPD-1 antibody compared to an αPD-1 antibody alone. C57BL/6 mice were injected subcutaneously with MC38i, an aggressive MC38 clone. When mean tumor volume ranged between 75–100 mm^3^, mice were randomized into groups of 5 and treated with indicated test articles at day 0 and 4 at 15 mg/kg. **(B)** C57BL/6 mice were injected subcutaneously with MC38i. When mean tumor volume ranged between 75–100 mm^3^, mice were randomized into groups of five and treated with indicated test articles at day 0 and 4 at 15 mg/kg. 100-fold (red) and 10,000-fold (blue) attenuated variant showed comparable anti-tumor efficacy. One mouse of the group treated with RMP-IL-18mutE4 was excluded for visual purposes but was included in the statistical analysis. The tumor growth curves of all individual mice are shown in **Supplementary Figures 4B-E**. **(C)** For the 100-fold attenuated variant, significant loss of weight was observed within one day after treatment. For the 10,000-fold attenuated variant, the change of body weight was comparable to the PBS control and PD-1 alone treatment. **(D)** TRs were rechallenged after 90 days. Mice treated with the 10,000-fold attenuated variant (blue) showed superior TGI in comparison to those treated with the 100-fold attenuated variant (red). **(E)** Mice were treated with different doses of the 10,000-fold attenuated RMP-IL-18mutE4. A dose-dependent effect was observed, where the treatment with 5 mg/kg showed compromised efficacy in comparison to the treatment with either 10mg/kg or 15mg/kg. **(F)** Treatment with the 10,000-fold attenuated variant targeted to PD-1 showed superior TGI in comparison to the treatment with an anti-IL-18BP antibody. Statistical significance was analyzed using a two-way ANOVA test.

Having shown the enhanced activity of the 10,000-fold attenuated IL-18 antibody fusion *in vitro*, we investigated *in vivo* anti-tumor activity of three IL-18mut variants fused to a single armed RMP1–14 characterized by attenuations of 100-fold up to 1,000,000-fold. An aggressive variant of MC38 colon carcinoma tumor model termed “MC38i” was employed to analyze the effect of different degrees of attenuation *in vivo* (**Figures 3A, B**; **Supplementary Figures 4B-E**). To this end, 1x10^6^ MC38i tumor cells were injected subcutaneously into the flank of C57BL/6 mice. When tumor volume reached 75–100 mm^3^, 15 mg/kg of RMP-IL-18mutE2, RMP-IL-18mutE4, RMP-IL-18mutE6 (1,000,000-fold IL-18 attenuation) or 15 mg/kg αPD-1, were administered on day 0 and 4. The 1,000,000-fold attenuated IL-18, RMP-IL-18mutE6 led to ~50% tumor growth inhibition (TGI), about 2 fold superior to αPD-1 alone but was far less effective than the 10,000-fold attenuated RMP-IL-18mutE4, for which we observed a response in which the tumor volume was below caliper-based detection or “TRs” in four out of five mice.

RMP-IL-18mutE2 showed comparable efficacy to RMP-IL-18mutE4, but caused weight loss and high serum IFNγ levels, indicating the need for careful potency adjustment to avoid toxicity (**Figure 3C** and **Supplementary Figure 4A**). Immune memory was assessed by re-challenging TRs mice after 90 days. Mice treated with RMP-IL-18mutE4 showed a stronger memory response than those treated with RMP-IL-18mutE2 (**Figure 3D**). Given that IL-18 has been reported to induce effector T cell memory differentiation (39), stronger immune memory may have resulted from superior targeting of PD-1+ TpEX by RMP-IL-18mutE4 versus RMP-IL-18mutE2, further addressed in the discussion. Based on these results, the 10,000-fold attenuated variant (RMP-IL-18mutE4) was selected for further experiments. To analyze the dose-response relationship, 5 mg/kg, 10 mg/kg and 15 mg/kg of RMP-IL-18mutE4 were tested in a MC38i tumor model. 5 mg/kg showed appreciably lower efficacy than 10 mg/kg and 15 mg/kg and no weight loss was observed at any of the three dose levels (**Figure 3E**). Since 15 mg/kg is well within the accepted dose range for high PD-1 receptor occupancy (RO) *in vivo (*40), it was used for confirmatory studies in a variety of mouse tumor models. Recently, an alternative strategy to “de-inhibit” wild type IL-18 via neutralization of IL-18BP already present in the TME using an anti-IL-18BP antibody has emerged (41). Therefore, we evaluated RMP-IL-18mutE4 alongside an anti-mouse IL-18BP antibody in the aggressive MC38i tumor model. We did not observe any appreciable TGI in anti-mouse-IL-18BP treated mice. In contrast, >100% TGI was observed the RMP-IL-18mutE4 treated cohort with an 80% TR rate (**Figure 3F**).

**Figure 4:**
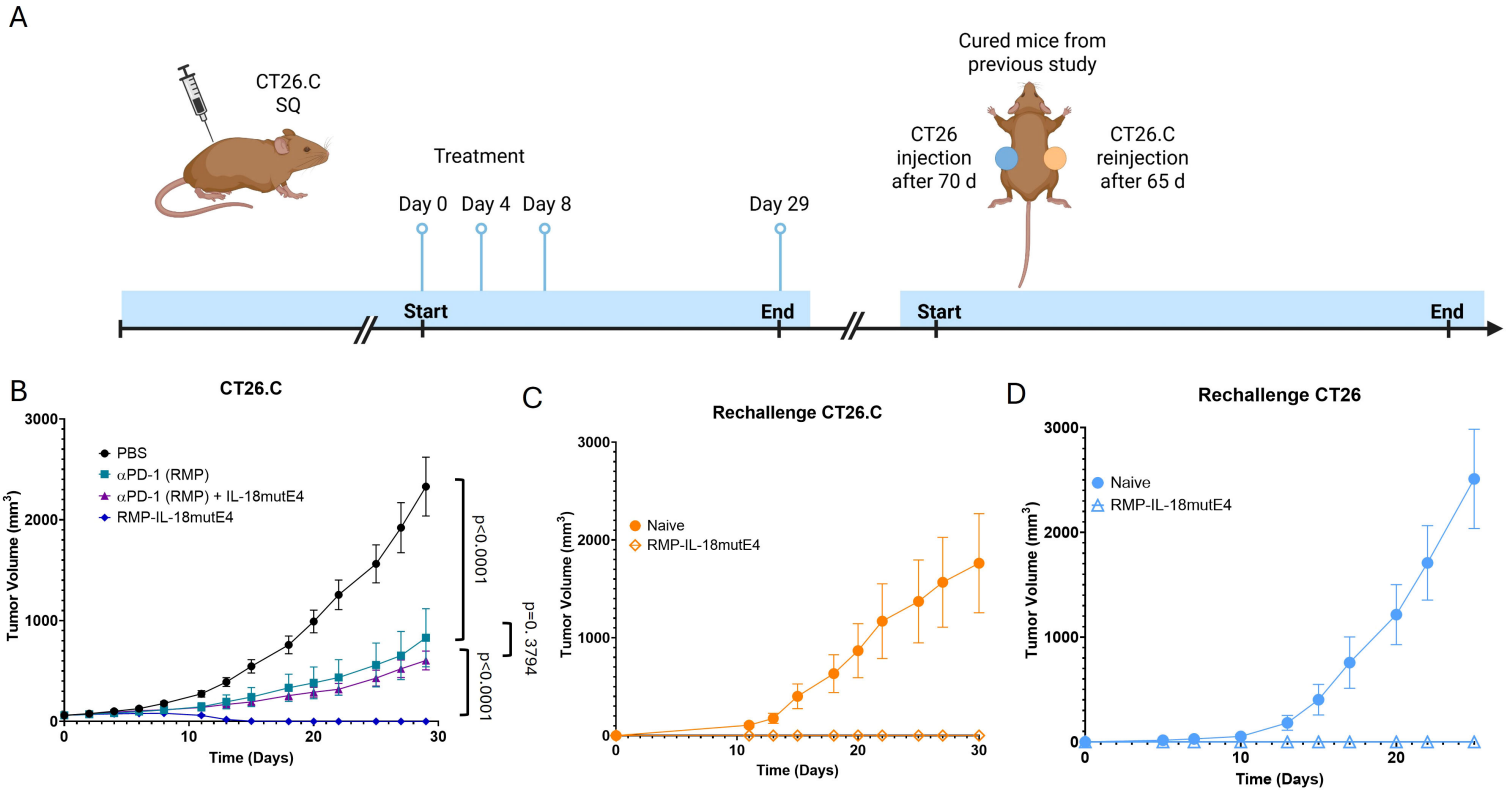
RMP-IL-18mutE4 induced memory and epitope spreading in an immunogenic colon carcinoma model. **(A)** TGI was analyzed in a CT26.C colon carcinoma tumor model. BALB/c mice were injected subcutaneously with CT26.C. When mean tumor volume ranged between 75–100 mm^3^, mice were randomized into groups of five. Treatment was conducted on day 0, 4 and 8 at 15mg/kg. **(B)** Tumor growth was monitored in CT26 tumor-bearing mice treated with the PD-1–IL-18mutE4 fusion protein (RMP-IL-18mutE4), αPD-1 (RMP) combined with attenuated IL-18mutE4, αPD-1 (RMP) alone, or PBS control. Mice treated with RMP-IL-18mutE4 were rechallenged after 65 days in the right flank with the same tumor line and after 70 days in the left flank with the parental tumor. No weight loss was observed. Statistical significance was analyzed using a two-way ANOVA test. **(C)** Rechallenge with the same tumor resulted in abolished tumor growth for the mice treated with RMP-IL-18mutE4. **(D)** Rechallenge with the parental tumor also resulted in complete TGI indicating epitope spreading induced by RMP-IL-18mutE4.

Next, RMP-IL-18mutE4 was evaluated in an immunogenic colon cancer model. BALB/c mice were injected subcutaneously with CT26.hCDH17 (CT26.C) tumor cells, representative of an immunogenic tumor expected to be responsive to αPD-1. TGI was analyzed for RMP-IL-18mutE4, αPD-1 alone, the combination of αPD-1 plus IL-18mutE4, and a PBS control group (**Figure 4**). 3*10^6^ CT26.C tumor cells were injected and when tumor volume reached 75–100 mm^3^, the drug was administered on days 0, 4 and 8 at 15 mg/kg. The combination of αPD-1 antibody and IL-18mutE4 was not significantly different compared to αPD-1 alone. RMP-IL-18mutE4 induced a markedly stronger TGI, with all mice exhibiting TRs (**Figure 4A**). Next, we rechallenged the cured mice with CT26.C on the left flank after 65 days and with parental CT26 on the right flank after 70 days. After 30 and 25 days, respectively, no tumor growth could be detected. In the reference group of five naive mice that received the same tumor inoculation, tumor growth to more than 1500 mm^3^ was observed (**Figures 4B, C**). That RMP-IL-18mutE4 treated and cured mice rejected CT26.C tumors is indicative of immune memory. Further, rejection of the parental tumor without the expression of immunogenic protein hCDH17 suggests that epitope spreading may have occurred, which implies that RMP-IL-18mutE4 may be able to treat and mediate durable responses against heterogenous tumors (42).

**Figure 5:**
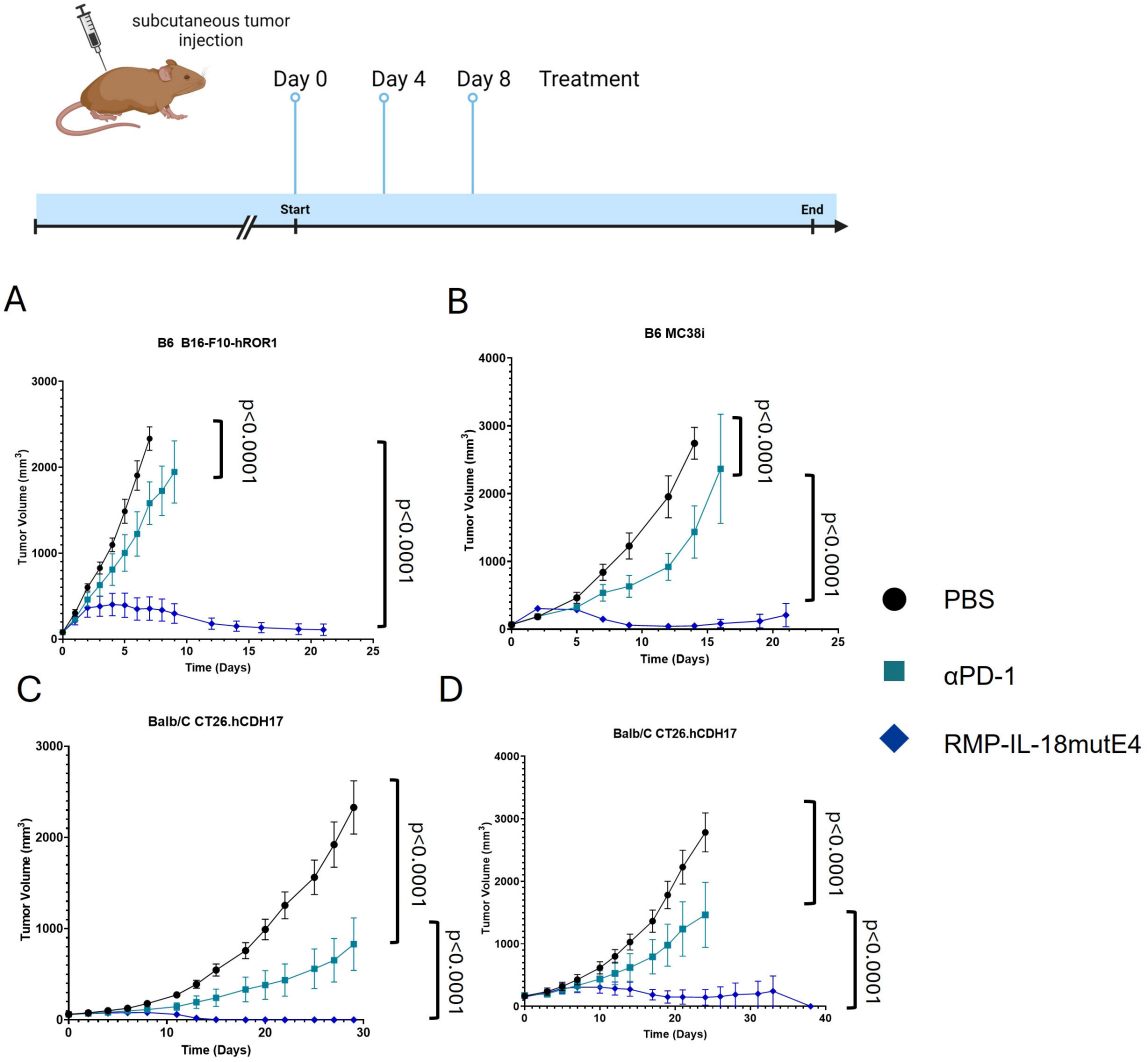
Analysis of anti-tumor effect of RMP-IL-18mutE4 in a variety of tumor models. Each group contained five mice. For each tumor model, there was one group that was treated with PBS, 15 mg/kg of αPD-1, or 15 mg/kg of RMP-IL-18mutE4. Treatment was conducted on day 0, 4, and 8. **(A)** TGI was analyzed in a B16-F10-hROR1 (B16-F10R) melanoma tumor model in B6 mice. Treatment was initiated when tumor volume reached 75 mm^3^. **(B)** TGI was analyzed in a MC38i tumor model in B6 mice. Treatment was initiated when tumor volume reached 75 mm^3^. **(C)** TGI was analyzed in a CT26.hCDH17 colon carcinoma tumor model in BALB/c mice. Treatment was initiated when tumor volume reached 75 mm^3^. **(D)** TGI was analyzed in a CT26.hCDH17 tumor model in BALB/c mice. Treatment was initiated when tumor volume reached 300–400 mm^3^. Statistical significance was analyzed using a two-way ANOVA test.

RMP-IL-18mutE4 was then tested in the following tumor models: B16-F10-hROR1 (melanoma), MC38i (colon adenocarcinoma), and CT26.hCDH17 (colon carcinoma) (**Figure 5**). All tumors were injected subcutaneously. In all tumor models, RMP-IL-18mutE4 was associated with appreciably superior TGI versus αPD-1 alone. RMP-IL-18mutE4 cured mice bearing CT26.hCDH17, B16-F10-hROR1, and MC38i tumors.

### 3.4 RMP-IL-18mutE4 induced strong lymphocyte infiltration of murine melanoma tumors

**Figure 6:**
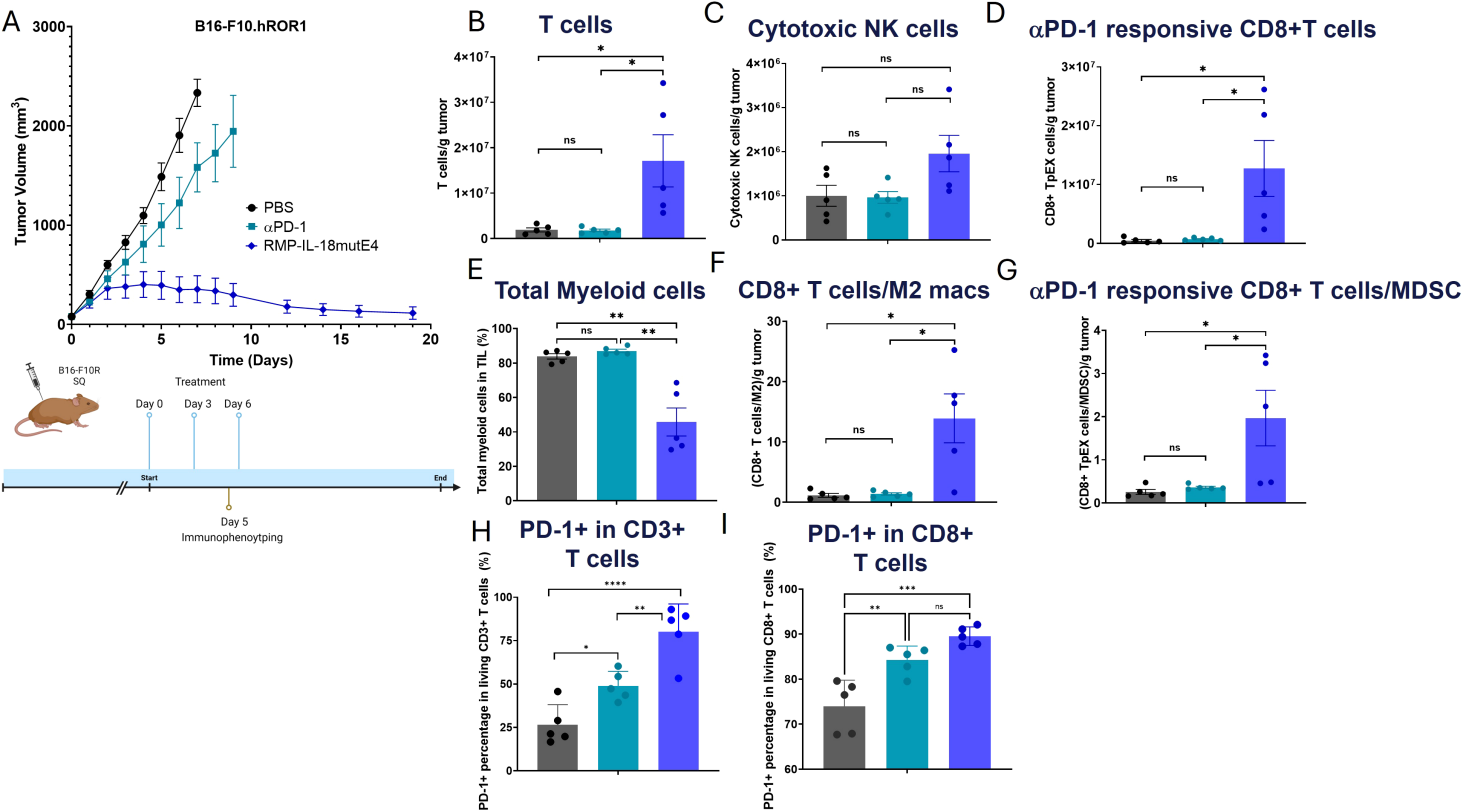
Strong infiltration of lymphocytes is induced by RMP-IL-18mutE4 in a B16-F10-R tumor model. Each group contained five C57BL/6 mice. Treatment was initiated when tumor volume reached 75 mm^3^ and conducted on day 0, 3 and 6 at 15 mg/kg. Immunophenotyping was carried out on day 5 by euthanizing the mice, tumor collection and dispersion into single cell suspensions for flow cytometric assessment of TIL (CD45 + cells). All myeloid subsets were gated on CD11b. Within that pool, MDSC were defined as Ly6G+/Ly6Cmid+Ly6G-/Ly6Chigh and M2 macrophages as F480+/CD206+. **(A)** Treatment with RMP-IL-18mutE4 resulted in effective TGI. Mice in the PBS group were euthanized after seven days and mice in the αPD-1 group were euthanized after nine days. **(B)** RMP-IL-18mutE4 induced a strong infiltration of T cells, whereas αPD-1 treatment showed no significant difference from the PBS group. **(C)** Comparison of cytotoxic NK cell infiltration. Cytotoxic NK cells were gated as CD11b+/F480-/Ly6C-low/mid/Ly6G-/MHC-II-. **(D)** RMP-IL-18mutE4 induced a strong infiltration of αPD-1 responsive CD8+ T cells, whereas αPD-1 treatment showed no significant difference from the PBS group. **(E)** RMP-IL-18mutE4 induced reduced presence of total myeloid cells, whereas αPD-1 treatment showed no significant difference from the PBS group. **(F)** RMP-IL-18mutE4 induced an increased CD8+ T cells to M2 macrophages ratio, whereas αPD-1 treatment showed no significant difference from the PBS group. **(G)** RMP-IL-18mutE4 induced an increased αPD-1 responsive CD8+ T cells to myeloid-derived suppressor cells (MDSCs) ratio, whereas αPD-1 treatment showed no significant difference from the PBS group. **(H)** Frequency of PD-1+ in CD3+ T cells. **(I)** Frequency of PD-1+ in CD8+ T cells. Statistical significance was analyzed using a one-way ANOVA test (ns P>0.05; *P ≤ 0.05; **P ≤ 0.01).

Next, we investigated the impact of RMP-IL-18mutE4 on the constitution of tumor infiltrating lymphocytes (TILs) in B16-F10R tumor bearing mice. Mice bearing tumors of 75–100 mm^3^ were randomized and treated with either PBS, 15 mg/kg αPD-1 or 15 mg/kg RMP-IL-18mutE4 on days 0, 3, and 6. Strong TGI was observed in the RMP-IL-18mutE4 treated group with three out of five showing TRs (**Figure 6A**). Tumor growth was rapid in both PBS and αPD-1 treated mice, which had to be euthanized after seven days in the PBS group and nine days in the αPD-1 group. To assess the infiltration of lymphocytes into tumors, five mice from each group were euthanized on day 5 and the tumor was harvested and dispersed into single cell suspensions for flow cytometric assessment (**Supplementary Figure 5**). Compared to PBS and αPD-1 treated animals, total T cells, CD8+ T cells, CD8+ central memory T cells, and CD8+ effector memory T cells were significantly increased in tumors from RMP-IL-18mutE4 treated mice (**Figures 6B-G**). To gain granularity on the impact of RMP-IL-18mutE4 on TIL exhaustion, we examined the cells for the expression of TCF-1, PD-1 and Tim3. The vast majority of T cells in the TME were PD1+ (**Figures 6H, I**), indicative of an antigen experienced state. Co-expression of PD-1 and TCF-1 on CD8+ T cells (the TpEX subset) has been shown to define the T cell population that is reinvigorated and responsible for the majority of αPD-1 mediated anti-tumor activity (6). Mice treated with RMP-IL-18mutE4 had lower amounts of tumor associated myeloid cells (tumor associated macrophages [TAM] and myeloid-derived suppressor cells [MDSC]), which play an important immunosuppressive and pro-metastatic role in tumor tissue via multiple pathways including PD-L1 dependent CD8+ T cell exhaustion, secretion of TGFβ and VEGF, and nitration of chemokines and TCRs (43–45). Treatment with RMP-IL-18mutE4 resulted in an elevated ratio of CD8+ T cells to M2 macrophages and of progenitor exhausted CD8+ T cells to MDSCs. These results indicate that RMP-IL-18mutE4 induces a robust immune response and induces the differentiation and proliferation of lymphocytes with anti-tumor activity.

## 4 Discussion

Overcoming resistance to checkpoint inhibition is a critical need in oncology. Resistance mechanisms include loss of MHC-I expression (46), altered interferon signaling (47), and an immunosuppressive TME involving the accumulation of MDSCs, M2 macrophages, and Tregs (48). Another critical barrier is T cell exhaustion (10). Combining pro-inflammatory cytokines with PD-1/PD-L1 inhibitors can counter T cell exhaustion and boost anti-tumor activity. However, cytokines, due to their potent but transient activity and short-range effects, face safety limitations and can induce exhaustion when used in traditional therapies that prioritize chronic systemic exposure (49). The goal of fusing a cytokine to an αPD-1/PD-L1 antibody is to generate a bifunctional molecule that delivers the pro-inflammatory activity of the cytokine while maintaining checkpoint inhibition. Effective inhibition requires sustained high PD-1/PD-L1 occupancy in the TME and draining lymph nodes for prolonged periods, but this is challenging due to cytokine potency and the pharmacokinetics (PK) of the fusion molecule.

The key subset of T cells responsible for the efficacy associated with anti-PD-1 checkpoint inhibitors are PD-1 positive progenitor exhausted CD8+ T cells (TpEX). These cells retain proliferative capacity and avoid terminal exhaustion via expression patterns of transcription factors and late-stage checkpoint receptors including maintenance of T-bet and TCF-1, low levels of Eomes and FOXO1, and avoidance of TOX and Tim3 (4, 25, 50). Therefore, a cytokine whose receptor is preferentially expressed on TpEX and whose signaling opposes terminal exhaustion via support of the TpEX expression pattern would be the ideal candidate for delivery to PD-1. Timperi and colleagues identified the IL-18R complex and IL-18 responsiveness as markers of the TpEX pool that they denoted as “functional CD8+ T cells” in TIL from NSCLC (51). Chmielewski and Abken screened 14 cytokines including IL-2 as candidates for expression by CAR-T cells and identified only IL-18 as capable of simultaneously strongly upregulating T-bet and downregulating FOXO1 (52).

Using the MC38 mouse tumor model, we screened an array of IL-18 variants fused to anti-mPD-1 (RMP1-14) and identified an optimal IL-18 variant which, relative to wild type mouse IL-18, is ~10,000-fold attenuated and therefore named RMP-IL-18mutE4. RMP-IL-18mutE4 showed strong tumor growth inhibition (TGI) including an average 85% TR rate across a variety of mouse tumor models. TRs were visually/microscopically confirmed during necropsy but confirmation and designation of complete responses using fluorescent and/or bioluminescent imaging would have added strong corroboration and represents a limitation of the study. RMP-IL-18mutE4 induced immunological memory and epitope spreading, restructured the TME to a highly elevated ratio of CD8+ TpEX to MDSCs, and critically could be dosed to levels up to 25 mg/kg without toxicity (**Supplementary Figure 6**). Beyond body-weight loss and systemic IFNγ release, IL-18mutE2-treated animals also showed visible signs of systemic inflammation, including splenomegaly, hepatomegaly, hunched posture, ruffled fur, reduced food and water intake, and lethargy. Although these observations support the higher systemic potency and toxicity of IL-18mutE2, a more comprehensive toxicological assessment will be necessary in future preclinical development. Our flow cytometry panel focused on major T-cell subsets and did not include B-cell or plasma-cell markers. Prior work in MC38 and related models indicates that antitumor activity of anti-PD-1 and PD-1 cytokine fusions is largely driven by T, NK, and myeloid cells rather than B-cell lineages (53). Future studies using expanded immunophenotyping panels will allow dedicated assessment of B-cell and plasma-cell populations, as well as deeper profiling of T-cell subsets. Notably, we did not perform a detailed comparison of downstream signaling cascades activated by the different IL-18 attenuation variants. Given that IL-18mutE6 is approximately 1,000,000-fold attenuated compared to the ~10,000-fold attenuated IL-18mutE4, it is likely that its markedly reduced intrinsic activity accounts for its lower antitumor efficacy. Future studies assessing pathway activation across IL-18 mutants will be important for defining the relationship between attenuation level and *in vivo* potency.

Interestingly, we observed stronger MC38 tumor-protective immune memory with RMP-IL-18mutE4 than RMP-IL-18mutE2, highlighting IL-18’s complex role in systems with variable PD-1 and IL-18R expression on T cells and myeloid subsets (54). In MC38 tumors, where most TILs are myeloid, PD-1 targeting by RMP-IL-18mutE4 is likely more T cell-restrictive. Less restricted targeting and trans activity from potent IL-18 drive myeloid-dependent anti-tumor responses, including M2-to-M1 macrophage repolarization, before T cell memory develops (55). IL-18-activated myeloid cells secrete NO, aiding tumor killing but potentially nitrating tumor-specific TCRs, impacting memory differentiation (56). PD-1’s role in tumor-induced emergency myelopoiesis is, in our opinion, underappreciated. Strauss et al. found PD-1 on myeloid subsets from progenitors to fully differentiated cells, and PD-1 knockout in myeloid cells was more effective against MC38/B16 tumors than knockout of PD-1 in T cells (57). Importantly, IL-18 has been shown to convert regulatory myeloid cells into effector phenotypes and enhance antigen presentation (52, 58). We hypothesize αPD-1 IL-18 fusion at high PD-1 RO could synergistically reprogram emergency myelopoiesis and enhance PD-1+ NK cell activity against MHC-I-deficient tumors, a resistance mechanism to αPD-1/L1 therapy. Supporting this, we observed increased cytotoxic NK cells in TILs of RMP-IL-18mutE4-treated mice, aligning with Chmielewski et al. (52).

In summary, we engineered an IL-18-BP-resistant IL-18 that regulates binding to IL-18Rα and restores IL-18 activity when delivered in cis via an IL-18mut-αPD-1 antibody fusion to IL-18R complex expressing PD-1+ cells. It remains to be elucidated whether a similar construct targeting human PD-1 with comparable intrinsically attenuated activity can mediate enhanced T cell anti-tumor activity and oppose functional exhaustion in humans.

## Data Availability

The raw data supporting the conclusions of this article will be made available by the authors, without undue reservation.
