# Supplementary material for: Targeting highly attenuated IL-18 to PD-1 for enhanced anti-tumor activity
Source: Front Immunol. 2025 Dec 18;16:1718321. doi: 10.3389/fimmu.2025.1718321 (PMC12756081; doi:10.3389/fimmu.2025.1718321)
Supplement: Supplementary file 1 [file DataSheet1.docx]

Supplementary Material

Targeting highly attenuated IL-18 to PD-1 for enhanced anti-tumor activity

**Xueyuan Zhou^1^**^+^**, Felix Klaus Geyer^2^**^+^**, Jeffrey Takimoto^1^, Harald Kolmar^2,3^*, Brian Rabinovich^1^***

1 Drug Discovery and Development, Fuse Biotherapeutics, Woburn, MA, United States

2 Institute for Organic Chemistry and Biochemistry, Technical University of Darmstadt, Darmstadt, Germany

3 Centre for Synthetic Biology, Technical University of Darmstadt, Darmstadt, Germany

^+^ These authors have contributed equally to this work and share first authorship.

*** Correspondence:**Brian Rabinovich

brabinovich@fusebiotx.com

Harald Kolmar

Harald.Kolmar@TU-Darmstadt.de

Supplemental Fig S1: HEK-BlueTM IL-18 assay of different IL-18mutants with different cysteine replacements (cysteine to serine AS or cysteine to valine AV) compared to wild type IL-18.


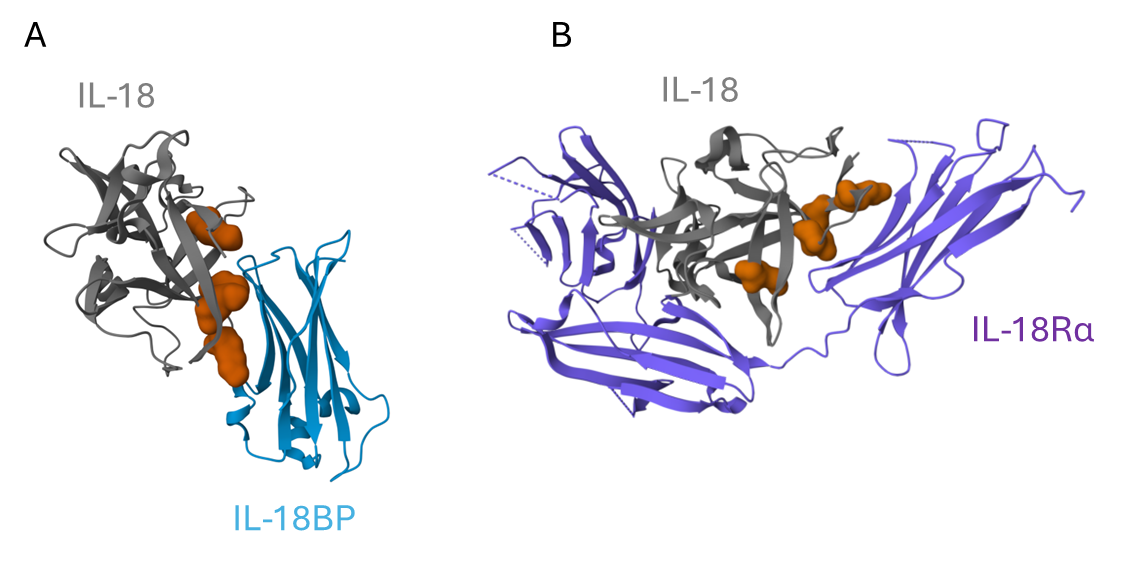


Supplemental Fig S2: Crystal structure of IL-18 (grey) in complex with the IL-18 BP (blue) (PDB 7AL7) and IL-18Rα (PDB 3WO3) (purple). Mutations introduced in IL-18 in the interface are highlighted in orange. (A) The mutations of IL-18mut2 in the IL18BP interface. (B) Crystal structure of IL-18 with highlighted mutations in the interface in complex with IL-18Rα.

Supplemental Fig S3: Analysis of IL-18 variants with different attenuation. CD3-primed mouse splenocytes were activated with 1 µg/mL anti-mouse CD3 antibody and afterwards incubated for 48 hours with the corresponding construct concentration and released mIFNγ was quantified by an ELISA assay.


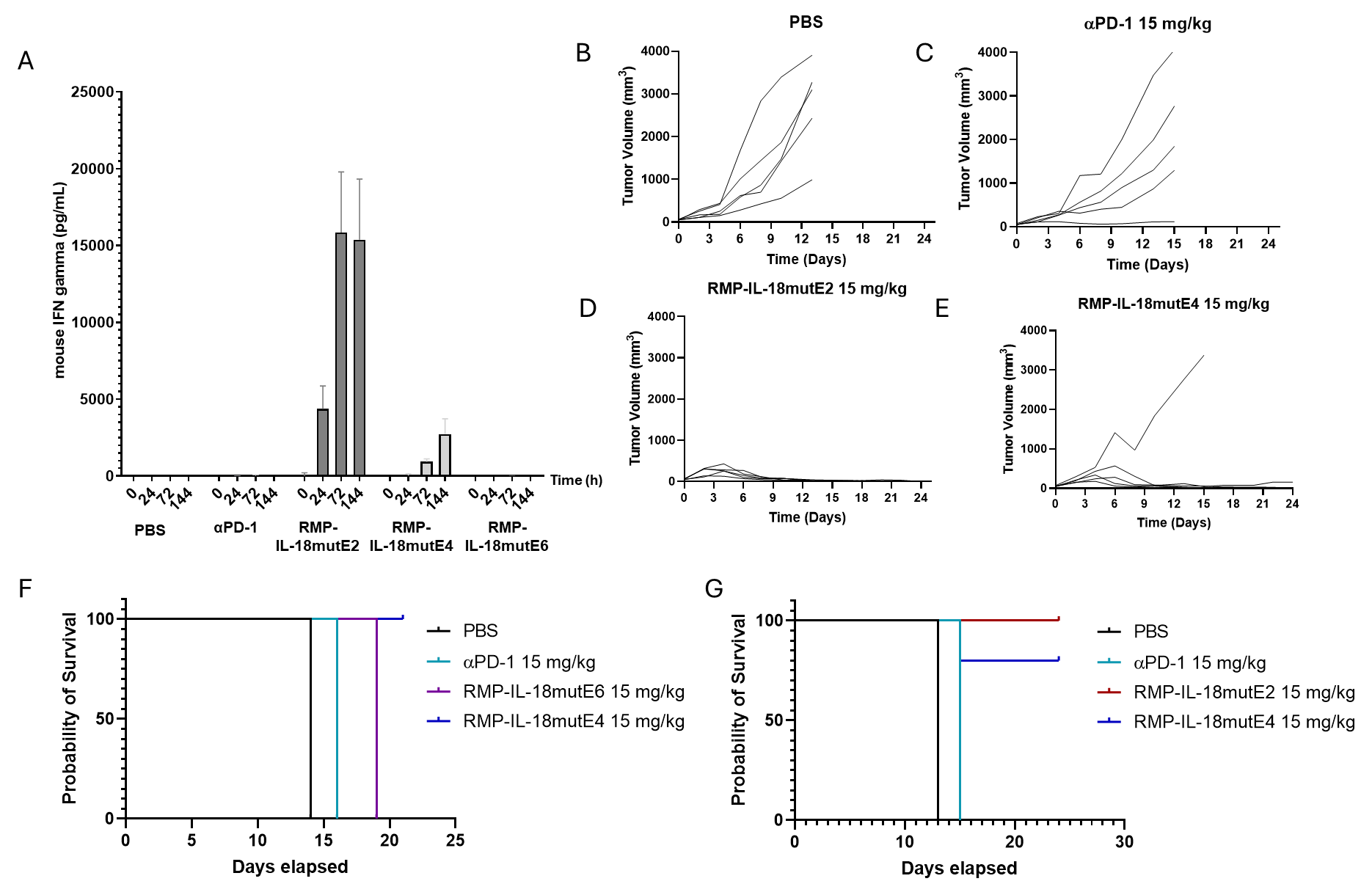


Supplemental Fig S4: Measurement of released mouse IFNγ in and individual tumor growth curves MC38i (anti-PD1 resistant MC38 clone) tumor bearing C57BL/6 mice. (A) Measurement of released mouse IFNγ after treatment with PBS, αPD-1, RMP-IL-18mutE2, RMP-IL-18mutE4, or RMP-IL-18mutE6 at a dose of 15 mg/kg (n=4). IFNγ was measured after 0, 24, 72, and 144 hours. (B) Tumor growth curves of the control group treated with PBS. (C) Tumor growth curves of the group treated with 15 mg/kg αPD-1. (D) Tumor growth curves of the group treated with 15 mg/kg RMP-IL-18mutE2. (E) Tumor growth curves of the group treated with 15 mg/kg RMP-IL-18mutE4 (n=5). (F) Kaplan–Meier survival curve corresponding to the treatment groups shown in Fig. 3A. (G) Kaplan–Meier survival curve corresponding to the treatment groups shown in Fig. 3B.


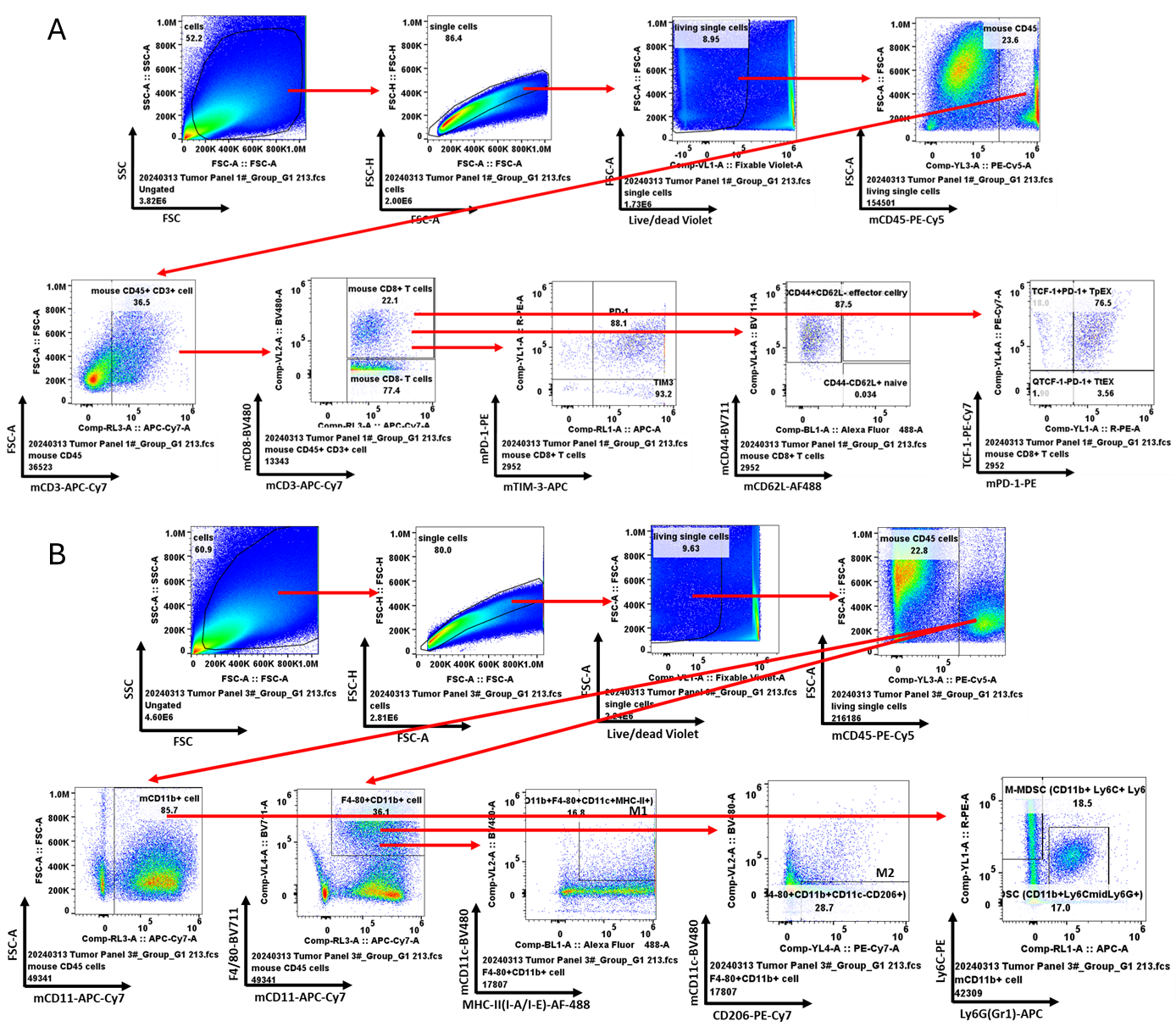


Supplemental Figure S5: Gating strategy for immunophenotyping analysis. (A) Gating strategy to analyze different lymphocyte populations. (B) Gating strategy to analyze different myeloid cell populations.


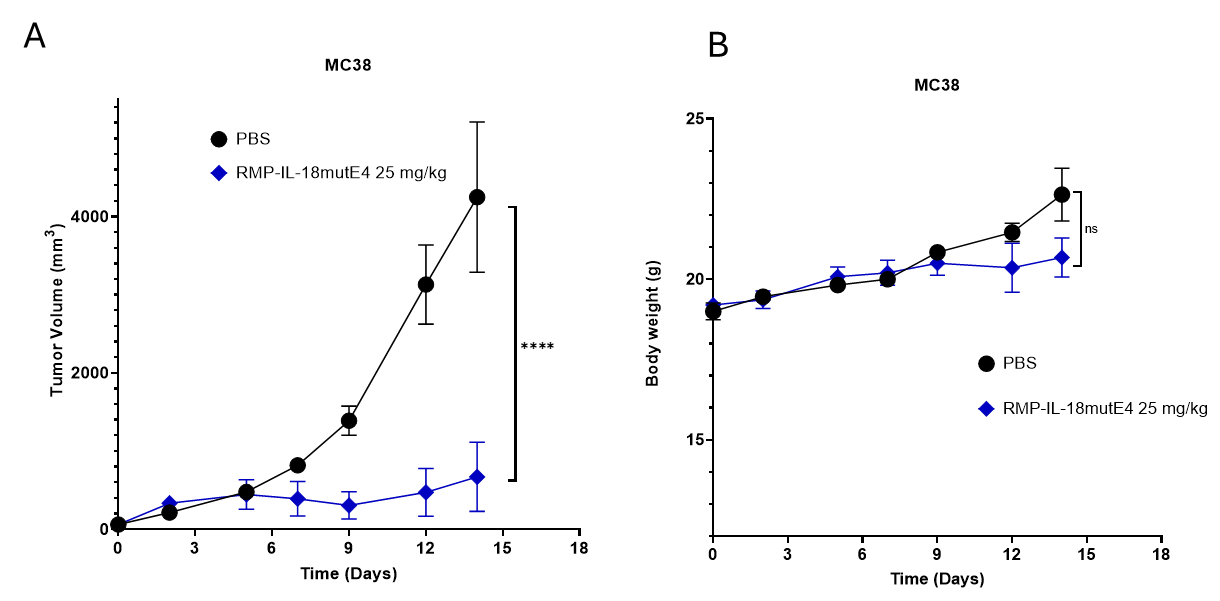


Supplemental Fig S6: (A) Treatment with RMP-IL-18mutE4 resulted in significant inhibition of tumor growth compared to the PBS control group. (B) No significant difference was observed for the body weight between the PBS control group and the group, which was treated with 25 mg/kg RMP- IL-18mutE4. Statistical significance was analyzed using a two-way ANOVA test (ns P>0.05; **** P≤0.0001).
